# Gaps in awareness of peripheral arterial disease in Sri Lanka: a cross sectional study

**DOI:** 10.1186/s12889-016-3748-8

**Published:** 2016-10-12

**Authors:** Janaka Weragoda, Manuj C. Weerasinghe, Rohini Seneviratne, S. M. Wijeyaratne

**Affiliations:** 1 Public Health Complex, 6th Floor, 555/5, Ministry of Health, Elvitigala Mawatha, Narahenpita, Colombo 10100 Sri Lanka; 2 Department of Community Medicine, Faculty of Medicine, University of Colombo, No.25, Kynsey Road, Colombo, 008000 Sri Lanka; 3 Department of Surgery, Faculty of Medicine, University of Colombo, No.25, Kynsey Road, Colombo, 00800 Sri Lanka

**Keywords:** Public awareness, Peripheral arterial disease, Cardiovascular disease, Cross sectional study, Sri Lanka

## Abstract

**Background:**

Peripheral arterial disease (PAD) is an emerging problem in Sri Lanka, particularly with the ageing population. A considerable number of patients are detected at a late stage with severe limb ischemia or chronic non-healing leg ulceration. Public awareness about PAD is important in developing preventive strategies.

**Methods:**

A cross sectional study was conducted to assess awareness of PAD among adults aged 40–74 years in a district in Sri Lanka. In total, 2912 adults were selected for the study using a multistage probability proportionate to size sampling technique. Data were collected by an interviewer-administered questionnaire. Participants who were aware of PAD were asked about common risk factors, possible consequences of untreated PAD, and sources of information. Multivariate logistic regression analysis was used to assess the independent predictors of PAD awareness.

**Results:**

We found that 4.1 % of participants were aware of PAD (95 % confidence interval: 3.4–4.8), which was significantly lower than awareness of other cardiovascular diseases such as cerebrovascular accidents (67.3 %) and myocardial infarction (57.6 %) (*p* < 0.001). Being male, an urban resident, and having a higher level of education were independent predictors of high PAD awareness.

**Conclusions:**

Our findings suggest that a comprehensive PAD awareness program that covers risk factors, consequences, and preventative strategies is needed to enhance public awareness of PAD.

## Background

Peripheral arterial disease (PAD) is one of the most common cardiovascular diseases in developed countries [1] and is an emerging problem in developing countries [2, 3]. The Trans-Atlantic Inter-Society Consensus reported the prevalence of PAD in European population studies ranged from 3.6 to 9.2 %, and was 10–20 % in those aged over 70 years [4]. A recent study in Sri Lanka found the age- and sex-standardized prevalence of PAD was 3.6 %, and there was significant increasing trend with age [3]. In the early stages, PAD is mostly silent. With the progression of disease, it may manifest as intermittent claudication, pain at rest, and frank tissue death resulting in lower-extremity amputation [5]. PAD is a major cause of disability, loss of employment, and lifestyle changes, and is a marker for systemic atherosclerotic diseases. The main risk to a patient experiencing claudication is a fatal or non-fatal cardiovascular event [4]. Despite the major health risks associated with PAD, it is generally not recognized by clinicians or the general public in comparison with other cardiovascular diseases. Most individuals with lower extremity PAD are asymptomatic and do not experience recognizable ischemic symptoms until late in the disease progression. However, asymptomatic individuals also have a higher risk of adverse cardiovascular events, similar to those with symptomatic PAD [6].

Low awareness makes addressing the public health impact of PAD challenging [7]. Many studies have shown that public awareness of PAD is much lower than that of other diseases. It has been reported that awareness of PAD ranged from 20 to 36 %, whereas awareness of other common diseases was more than 60 % in the same population [6–9]. Awareness is important for patients and physicians, and the need for public awareness programs has been highlighted [10, 11]. Thereare no published literature on awareness of diseases in Asian countries.

Although the prevalence of PAD in Sri Lanka is not as high as in Europe or the United States, there is a consistent age-related trend [3]. Anecdotal evidence suggests that a considerable number of patients are detected in the late stage of PAD with severe limb ischemia or chronic non-healing leg ulceration. It is difficult to reduce the morbidity and mortality of untreated PAD without adequate public awareness of PAD and its risk factors and consequences [7]. Insights into public awareness of PAD will help in developing strategies for behavioral change communication and health promotion. In this study we aimed to assess awareness of PAD among adults aged 40–74 years in a district in Sri Lanka.

## Methods

### Study population

We conducted a cross sectional study to assess awareness of PAD in adults as part of a prevalence study in Gampaha district, Sri Lanka from 2012 to 2013 [3]. Gampaha is the second most populous district in Sri Lanka, and has 2 million inhabitants. Gampaha district is divided into 13 divisional secretariat areas for administrative purposes. We used a multistage probability proportionate to size sampling technique to recruit 2912 adults aged 40–74 years from 104 clusters in four randomly-selected divisional secretariat areas in Gampaha.

The sample size was determine using the formula for prevalence study described in Lwanga and Lemeshow [12]. Anticipated population prevalence of PAD was assumed to be 3.5 % with 1 % precision required on either side of the proportion. The required sample size was corrected to account for the cluster sampling methodology and for non-response among participants. Thus, the sample size required to detect the expected prevalence in the community with 95 % confidence interval and with 1 % precisionwas 2912.

A cluster was defined as an administrative area of a village officer, and cluster size was 28. The area of a village officer is the smallest administrative unit in Sri Lanka. The age range (40–74 years) was divided into 5-year age groups, and a sample was obtained from each cluster. The number of individuals included in each age group was based on the proportion of the population in each age group in the 2001 national census data. An equal number of males and females were selected for each age group from each cluster. A detail description of the sampling method is published elsewhere [3].

### Data collection

The study instrument was an interviewer-administered questionnaire. Questionnaire development was based on several previous studies [6, 7] with the help of an expert panel. Sociodemographic characteristics such as age, sex, level of education, and monthly household income were collected. Age was verified by supportive documents. House to house survey was carried for Data collection. Respondents were interviewed in their own residences including the clinical measurements of ankle brachial pressure index (ABPI) to detect PAD. Arterial Doppler instrument was used to measure the ABPI. For more details, see Weragoda et al. 2015 [3]. Self-reported information on medical history of diabetes mellitus (DM), dyslipidemia, and hypertension was obtained and verified by available clinical records or medications. Information on smoking was also obtained, with exposure categorized according to the classification of the Centers for Disease Control and Prevention in the United States [13]. Assessment of intermittent claudication was based on the Edinburgh Claudication Questionnaire [14].

Participants’ awareness was classified in three groups: “not heard about the disease,” “heard but could not describe the disease,” and “heard about and could describe the disease.” As participants were unfamiliar with the English term PAD, the disease was explained as “blocked or narrowing of arteries of the legs, affecting blood circulation.” Awareness of cerebrovascular accidents (CVA) and myocardial infarction (MI) was assessed in a similar way. CVA was explained as “stroke” (“Anshabhagaya” in the Sinhala language) and MI was explained as “heart attack,” which was the terminology commonly used by the public. Participants who were aware of PAD were asked about common risk factors, possible consequences of untreated PAD, and their sources of information.

The Ethics Review Committee of the Faculty of Medicine, University of Colombo granted approval for the study (Protocol No. EC-12-13). Informed consent was obtained from all participants before participation in the study.

### Data analysis

In the analysis, participants who “had not heard about the disease” and ‘heard but could not describe the disease” were combined into one group “not aware” of the disease (PAD, CVA, or MI). Those who had “heard about and could describe the disease” were classified as “aware” of that disease. Participants’ awareness of PAD was compared with awareness of other atherosclerotic diseases (MI and CVA) using paired-samples t-tests. Bivariate analysis for awareness of PAD by selected participant characteristics was performed, and differences between groups were analyzed with Pearson’s chi-square tests. Multivariate logistic regression analysis was used to assess the independent predictors of awareness of PAD. The independent factors included in the model were various sociodemographic measures, presence of PAD risk factors such as DM, hypertension, dyslipidemia, smoking, and the presence of intermittent claudication. We used SPSS Version 16 (SPSS for Windows, Version 16.0. Chicago, SPSS Inc.) for the analyses. Statistical significance was set at *p* < 0.05.

## Results

We recruited 2779 participants from the selected sample of 2912 eligible participants. The response rate was 95.4 %. The proportion of male and female participants was almost equal. The median age of respondents was 52 years (IQR 46–61 years). The awareness of PAD was 4.1 % (95 % confidence interval: 3.4–4.8), which was significantly lower than that of CVA (67.3 %) and MI (57.6 %) (*p* < 0.001) (Table 1).

**Table 1:** Awareness of peripheral arterial disease, cerebrovascular accidents, and myocardial infarction (*n* = 2779)

Disease	Aware		Not aware ^a^		Significance (paired *t*-test)
	No.	%	No.	%	
PAD (Blocked arteries in lower limbs)	114	4.1	2665	95.9	Reference
CVA (Stroke)	1869	67.3	910	32.7	*p* < 0.001
MI (Heart attack)	1600	57.6	1179	42.4	*p* < 0.001

*CVA* cerebrovascular accident, *MI* myocardial infarction, *PAD* peripheral arterial disease

^a^ “not heard about the disease” and “heard but could not describe the disease” combined as “not aware”

The awareness of PAD by participant characteristics is described in Table 2. There were no significant differences in awareness of PAD by age group or level of income (*p* > 0.05). In addition, there were no significant differences in awareness of PAD among current smokers, those who had intermittent claudication, or those with a history of DM, hypertension, or dyslipidemia (*P* > 0.05). Multivariate logistic regression analysis showed that being male, an urban resident, and having a higher education level (General Certificate of Education Ordinary Level or above) were independent predictors of high awareness of PAD.

**Table 2:** Association between awareness of peripheral arterial disease and participant characteristics (*n* = 2779)

Socioeconomic characteristics		Awareness of PAD				Total	Significance	
		Aware (*n* = 114)		Not aware ^a^ (*n* = 2665)			Bivariate analysis	Multiple logistic regression analysis
		No	%	No	%			
Age							*p* = 0.273	*p* = 0.387
40–49		47	4.0	1125	96.0	1172		
50–59		39	4.8	759	95.2	798		
≥60		28	3.5	781	96.5	809		
Sex							*p* < 0.001	*p* < 0.001
Male		73	5.2	1310	94.8	1383		
Female		41	2.9	1355	97.1	1396		
Sector in residence							*p* = 0.006	*p* < 0.001
Residence in urban		34	6.2	517	93.8	551		
Residence in rural		80	3.6	2148	96.4	2228		
Highest level of education							*p* < 0.001	*p* < 0.001
Up to GCE O/L		14	1.6	878	98.4	892		
GCE O/L completed and above		100	5.3	1787	94.7	1887		
Family income Rs.							*p* = 0.143	*p* = 0.760
<30,000		45	3.5	1238	96.5	1283		
≥30,000		69	4.6	1427	95.4	1496		
Current smokers		20	5.2	364	94.8	384	*p* = 0.191	*p* = 0.419
Presence of claudication symptoms		13	8.7	136	91.3	149	*p* = 0.088	*p* = 0.061
Medical history								
Diabetes mellitus	Yes	29	5.5	498	94.5	527	*p* = 0.717	*p* = 0.452
	No	85	3.2	2167	96.8	2252		
Hypertension	Yes	32	5.3	571	94.7	603	*p* = 0.091	*p* = 0.064
	No	82	3.1	2094	96.9	2176		
Dyslipidemia	Yes	26	5.2	472	94.8	498	*p* = 0.164	*p* = 0.579
	No	88	3.3	2193	96.7	2281		

*GCE O/L* general certificate of education ordinary level, *PAD* peripheral arterial disease

^a^ “not heard about the disease” and “heard but could not describe the disease” combined as “not aware of PAD”

The majority of participants who were “PAD aware” knew that DM (64.0 %) and dyslipidemia (57.9 %) were risk factors for PAD. However, less than one-third of this group was aware that hypertension (31.6 %) and smoking (30.7 %) were risk factors for PAD (Table 3). Walking difficulty and leg amputations were correctly identified as possible consequences of untreated PAD by more than 80 % of the PAD aware group. In contrast, less than 25 % were aware that CVA and MI were possible consequences of PAD (Table 4). In this group, the main sources of information about PAD were physicians (39.5 %) and family members or relatives (37.7 %) and only 10.5 % had received information from mass media such as newspapers, radio, and television (Table 5).

**Table 3:** Perceived risk factors for peripheral arterial disease according to “aware” participants (*n* = 114)

Risk factor	Increases the risk		Does not increase the risk		Do not know		Total	
	No	%	No	%	No	%	No	%
Diabetes	73	64.0	6	5.3	35	30.7	114	100
Dyslipidemia	66	57.9	10	8.8	38	33.3	114	100
Hypertension	36	31.6	12	10.5	66	57.9	114	100
Smoking	35	30.7	14	12.3	65	57.0	114	100
Obesity	35	30.7	05	4.4	74	64.9	114	100
Family history of PAD	51	44.7	11	9.6	52	45.6	114	100

*PAD* peripheral arterial disease

**Table 4:** Perceived consequences of untreated peripheral arterial disease according to “aware” participants (*n* = 114)

Perceived consequences	Yes		No		Do not know		Total	
	No	%	No	%	No	%	No	%
CVA	28	24.6	03	2.6	83	72.8	114	100
MI	21	18.4	05	4.4	88	77.2	114	100
Walking difficulty	98	86.0	--	--	16	14.0	114	100
Amputation	92	80.7	01	0.9	21	18.4	114	100

*CVA* cerebrovascular accident, *MI* myocardial infarction, *PAD* peripheral arterial disease

**Table 5:** Sources of information on peripheral arterial disease for “aware” participants (*n* = 114)

Source of information	No.	Percent
Physician/Family doctor	45	39.5
Family member/Relative/Friends	43	37.7
Newspapers	05	4.4
Radio	04	3.5
Television	03	2.6
Internet	03	2.6
Magazine	02	1.8
Field health workers	02	1.8
Could not give the source of information	07	6.1

*PAD* peripheral arterial disease

## Discussion

To our knowledge, this is the first study conducted in Sri Lanka to assess public awareness of PAD. In addition, awareness of two common atherosclerotic diseases (MI and CVA) was also assessed and compared with the level of awareness of PAD. Several studies conducted in the United States and Canada compared awareness of PAD with that of common diseases such as DM, hypertension, and dyslipidemia, as well as with some less common diseases such as multiple sclerosis and cystic fibrosis [6, 7].

In Sri Lanka response rate for household survey is generally very high. Potential participants rarely refuse to participate particularly in studies related to health. This was reflected in this study also with the response rate over 95 %. The population proportions﻿ in﻿ urban and rural divisions in the Gampaha district according to the last census in 2012 was 15.6 % and 84.4 % respectively [15]. Hence, majority in the study population belonged to the rural sector.

Our results showed that more than 95 % of participants were not aware of PAD. Different levels of PAD awareness have been reported by previous population-based studies in the United States (25 %) [6], Canada (36 %) [7] and the Netherlands (<20 %) [8]. Previous studies have also reported a significantly lower level of public awareness of PAD compared with other cardiovascular diseases such as CVA and coronary artery disease (60–75 %) [6, 7]. However, these previous studies were conducted in Europe or the United States and there are nopublished literatures on awareness of PAD in Asian countries.

We found that being male, an urban resident, and having a higher educational level were predictors of high awareness of PAD. However, those with a history of DM, hypertension, dyslipidemia, smoking, and leg symptoms of PAD did not show a significantly higher level of awareness of PAD. The literature shows that DM, hypertension, dyslipidemia, and smoking are well known as the greatest risk factors for PAD [16–19]. Therefore, there was a notable gap in awareness in those who are already at risk for PAD.

We also assessed the awareness of possible risk factors and consequences of PAD in the PAD aware group. We found less than one-third of this group was aware that hypertension and smoking were risk factors for PAD.A study in Ireland reported that more than 80 % of the study sample correctly identified smoking as a risk factor for PAD [9]. However, similar to our study, many other studies found that less than 50 % of participants were aware that smoking and hypertension were risk factors for PAD [6, 7]. Although it has been well identified that PAD is associated with an elevated risk of cardiovascular and cerebrovascular events and death from MI and CVA [20–22], we found that less than 25 % of our “PAD aware” group was aware that MI and CVA are possible consequences of untreated PAD. Many previous studies have also reported that less than 20 % identified CVA and MI as possible consequences of untreated PAD [6, 7]. These results highlight that even among those who are aware of PAD; overall understanding of PAD is poor. We found that very low proportion (10.5 %) of participants had received information on PAD from mass media. In contrast, studies in the United States and Canada reported that 31–33 % of those who were aware of PAD reported public media as their primary source of information [6, 7]. This suggests that information about PAD via public media is scarce in Sri Lanka.

Internationally, peripheral arterial disease or PAD is used in scientific writing and lay communication. Cronin et al. [9] attribute low awareness of PAD to the terminology not being familiar to the public. They highlighted the necessity of simple terminology for PAD to enhance public awareness. Lack of simple and consistent terminology for PAD also might have contributed to the low awareness of PAD in our study. Therefore, simple terminology to describe PAD is an ﻿emerging need﻿﻿.

## Conclusions

This study highlighted the low awareness of PAD in the general public in Sri Lanka. This low level of awareness may lead to delayed diagnosis, low compliance, and unfavorable outcomes for patients. This shows it is necessary to incorporate PAD to the major NCD prevention programme of the country to enhance public awareness of PAD, including risk factors and preventative methods. A simple terminology to describe PAD is needed to enhance public awareness of PAD﻿.

## Abbreviations

ABPI: Ankle brachial pressure index
CVA: Cerebrovascular accident
IAQ: Interviewer administered questionnaire
MI: Myocardial Infarction
MLR: Multiple logistic regression
PAD: Peripheral arterial disease
